# Periodontal Status and Quality of Life: Impact of Fear of Pain and Dental Fear

**DOI:** 10.1155/2017/5491923

**Published:** 2017-03-09

**Authors:** Casey D. Wright, Daniel W. McNeil, Cierra B. Edwards, Richard J. Crout, Katherine Neiswanger, John R. Shaffer, Mary L. Marazita

**Affiliations:** ^1^Department of Psychology, West Virginia University, 53 Campus Drive, P.O. Box 6040, Morgantown, WV 26506, USA; ^2^Center for Oral Health Research in Appalachia (COHRA), University of Pittsburgh, Pittsburgh, PA 15219, USA; ^3^Department of Dental Practice & Rural Health, Center for Oral Health Research in Appalachia (COHRA), School of Dentistry, West Virginia University, One Medical Center Drive, Morgantown, WV 26506, USA; ^4^Department of Periodontics, Center for Oral Health Research in Appalachia (COHRA), School of Dentistry, West Virginia University, One Medical Center Drive, Morgantown, WV 26506, USA; ^5^Department of Oral Biology, Center for Craniofacial and Dental Genetics, School of Dental Medicine, University of Pittsburgh, Bridgeside Point Suite 500, 100 Technology Drive, Pittsburgh, PA 15219, USA; ^6^Department of Human Genetics, Graduate School of Public Health, University of Pittsburgh, 130 De Soto Street, Pittsburgh, PA 15261, USA

## Abstract

*Background*. Oral health-related quality of life (OHRQoL) is impacted by periodontal disease and orofacial pain. There is a limited research examining the impact of avoidance of care or physiological arousal related to the fear of pain response on periodontal-related OHRQoL.* Methods*. Data are from the Center for Oral Health Research in Appalachia family-based study focusing on 1,339 adults. Measures included a modified Periodontal Screening and Recording Index across sextants of dentition, dental fear survey, Fear of Pain Questionnaire-9, and Oral Health Impact Profile-14. Structural equation modeling was used to estimate the effects of periodontal disease screening indicators on OHRQoL including the mediating role of dental fear while accounting for fear of pain.* Results*. A significant total effect was found for the mandibular anterior sextant, components of dental anxiety/fear, and indicators of OHRQoL (pain and discomfort, *β* = .165, *p* = .001; psychosocial impact, *β* = .199, *p* < .001). The maxillary anterior region was significantly associated with pain discomfort (*β* = .116, *p* = .017) and functionality (*β* = .130, *p* = .011).* Conclusions*. Findings provide a granular perspective of periodontal disease indicators and OHRQoL. Dental avoidance/anticipatory fear and physiological arousal mediate OHRQoL in individuals who have indicators of periodontal disease in sextants that may be visible and susceptible to higher pain and psychosocial impact.

## 1. Introduction

Chronic periodontal disease is defined as inflammation of the gingiva extending into the adjacent attachment apparatus. Clinical features may include edema, erythema, gingival bleeding upon probing and/or suppuration, and attachment loss [1]. Prevalence rates range from 25 to 54 percent in gingivitis (early disease with no attachment loss) to 43 percent with chronic periodontitis determined by one or more sites of attachment loss [2]. Established risk factors for developing periodontal disease include increasing age, tobacco use and alcohol consumption, genetic factors, obesity, poorly controlled diabetes, and psychological stress [3, 4]. Bouchard et al. [5] established a conceptual framework of risk for periodontal disease. The relations between risk and causal chains and causal networks leading to chronic periodontitis were highlighted, including a broad range of factors, ranging from distal ones (e.g., lifestyle) to biomarkers [5]. Additionally, research has indicated a link between localized periodontitis and enamel pearls suggesting that enamel pearls may be a causative factor, especially in maxillary molars [6]. Understanding chronic periodontitis as “among the most complex noncommunicable diseases” [5, p. 1] leads one to consider a wide range of factors, such as quality of life, that may provide additional insight into the clinical treatment and management of pain and disease.

Periodontal conditions often are not recognized in their early stages [7]. As periodontal disease progresses, signs such as swollen and bleeding gums and, ultimately, tooth mobility due to loss of support become apparent and possibly painful [8, 9]. A likely deterrent for managing such conditions could involve avoidance of brushing, flossing, or engaging in routine dental care. Due to its sequela, periodontal disease is a major cause of tooth loss in adults [10]. Although there is no evidence for causality, periodontal disease is associated with broader systemic health concerns including osteoporosis, rheumatoid arthritis, cardiovascular disease, and cognitive loss [11–13]. Women with periodontal disease also are more likely to have pregnancy complications [14].

In clinical periodontal applications, after a periodontal evaluation that includes probing depths and location of gingival margin (clinical attachment level), treatment includes removal of supragingival and subgingival plaque and calculus, as well as scaling and root planning. If treatment is not successful, antimicrobial agents or surgical treatment may be necessary [1]. Behavioral approaches are used to address long-term tertiary prevention, including oral hygiene instructions, adherence with suggested periodontal interventions, and counseling for control of risk factors (e.g., cessation of smoking and coping with stress) [1].

Although a variety of intervention methods exist, many individuals do not seek treatment for periodontal disease until it is at an advanced stage, in which tooth loss is common. Research is needed, therefore, at earlier periods when periodontal involvement is less advanced (e.g., gingivitis) and when screening indicators (e.g., attachment levels) may be the harbinger of more progressive disease (e.g., periodontitis). Additionally, few investigations have been directed to a precise understanding of periodontal status in regions of the oral cavity and their relation to quality of life or into the mechanisms of avoidant behavior and periodontal disease.

Periodontal disease has been linked to lower quality of life [15]. Quality of life refers to one's perceptions about life, both positive and negative, as a result of the cultural context in which one lives [16]. Quality of life encompasses several domains, including psychological functioning and social relationships among others. Oral health-related quality of life (OHRQoL, i.e., quality of life specific to oral health and the impacts of oral health) has been shown to be reduced in patients with periodontal disease [17–19]. Reduction in OHRQoL may be due to increased pain, tooth loss, and resultant loss of function. As an example, individuals with periodontal disease report negative impacts of their oral health on their overall functioning such as greater functional limitation, physical pain, and psychological discomfort [20]. Due to the deleterious effects of periodontal disease on health and quality of life, it is important to identify potentially modifiable factors that may mediate these relations. Understanding such indirect pathways may provide areas for future interventions to help alleviate the more direct negative effects on quality of life.

Given the greater burden of periodontal and other oral diseases in northern Appalachia relative to other regions and the general population in the USA [21], population samples from this region can provide an important basis to understand the relationship between oral diseases and OHRQoL and, in addition, allow assessment of additional psychosocial factors. Greater dental care-related anxiety and fear [22] are associated with poorer oral health status [23] possibly due, in part, to decreased care-seeking. Fears about pain during dental treatment have been identified as a major component of dental fear [24] and thus may indirectly affect dental treatment utilization. Mehrstedt et al. [25] and more recently Vermaire et al. [26] studied the effects of dental care-related fear and anxiety on quality of life and found that dental fear/anxiety contributes significantly to disease burden. Additionally, a study on the effects of dental anxiety/fear in quality of life in patients with temporomandibular disorders found that those with higher levels of dental anxiety/fear had poorer OHRQoL [27]. Given its prevalence across the population [22], including in north central Appalachia, dental care-related anxiety and fear are a particularly relevant psychosocial factor, along with fear of pain, that may affect the relation between periodontal disease and OHRQoL.

This rationale is supported by the documented association of dental fear and periodontal disease indicators. Ng and Leung [28] examined dental fear and its relation to periodontal clinical attachment levels and oral health-related quality of life. Greater levels of dental care-related anxiety and fear were associated with lower clinical attachment levels. Increased levels of dental fear also were related to poorer OHRQoL [28]. This relation was found in their sample of Hong Kong adults but has not been investigated in other population groups. Additionally, dental care-related anxiety and fear were treated as a single construct, while others assert the benefit of a more granular view [22]. Finally, the indirect link of fear of pain has yet to be explored in its relation to periodontal disease, dental fear, and OHRQoL.

The current study aimed to elucidate associations among fear of pain, dental fear, periodontal disease, and OHRQoL in the COHRA1 sample from north central Appalachia, associated with the Center for Oral Health Research in Appalachia (COHRA). More specifically, the direct and indirect effects of periodontal disease indicators on OHRQoL were investigated, while accounting for fear of pain and aspects of dental care-related anxiety and fear. There were two hypotheses. First, greater indications of periodontal disease (i.e., deeper PSR probing depths) in specific sextants were expected to be related to poorer OHRQoL. Second, it was hypothesized that components of dental care-related anxiety and fear, while accounting for fear of pain, would mediate the association between periodontal disease indicators and OHRQoL.

## 2. Methods

### 2.1. Participants

The COHRA1 protocol and sample have been previously described [29]. The COHRA1 project examined the genetic, psychosocial, and microbiological differences among families (comprising at least one parent-child pair) from rural West Virginia and Pennsylvania. Data were collected via interviews (i.e., demographic, oral health, and medical and family history), self-report questionnaires (e.g., health survey, dental fear survey, perceived stress, health locus of control, and nicotine dependence), and intraoral health assessments, which included periodontal probing among other disease indicators.

The present study utilized participants of age 18 and older with measures of periodontal probing depths, fear of pain, dental fear, and oral health-related quality of life. The present sample consisted of 1,339 individuals (63.6% females and 36.4% males) with a mean age of 34.3 (SD = 9.5; range = 18–93).  Table 1 describes participant characteristics. Consistent with demographics of this region of Appalachia, the participants were predominantly Caucasian (88.4%) but included 7.7% African Americans, 0.7% Hawaiian/Pacific Islanders, and 3.2% other racial/ethnic groups or missing.

### 2.2. Psychosocial Assessments

*Dental Fear Survey (DFS)*. The DFS is a 20-item, Likert-type, self-report instrument targeted at detecting individual differences related to dental fear [30]. The three subscales—avoidance and anticipatory fear, fear of specific dental stimuli, and physiological arousal—allow for a multilevel understanding of an individual's dental fear. The three subscales were used in this study to provide a more detailed analysis of dental care-related anxiety and fear. Various studies have been conducted to assess the psychometrics of the DFS [31, 32]. There is evidence for the internal consistency of the DFS (*α* = .80) [33] and data demonstrating overall construct validity [34, 35]. Furthermore, researchers have cross-validated the three-factor structure of the DFS in numerous cultural groups [36, 37].

*Fear of Pain Questionnaire-9 (FPQ-9)*. The FPQ-9 is a nine-item self-report questionnaire; the three subscales (fear of severe pain, fear of minor pain, and fear of medical pain) reflect the structure of the FPQ-9 parent instrument, the Fear of Pain Questionnaire-III [38]. Data from each of the FPQ-9 individual subscales and the total score can be interpreted to indicate adequate internal consistency (*α* = .84, severe pain; *α* = .72, minor pain; *α* = .73, medical pain; *α* = .83, total score). The FPQ-9 is scored by summing three item scores for each of the three subscales and a total score can be calculated by totaling all nine items. This study utilized the FPQ-9's higher order factor structure (three subscales loading onto a total score). The short instrument was shown to be highly correlated with the original FPQ-III (*r* = .94–.97) suggesting concurrent validity to the already validated FPQ-III [38].

*Oral Health Impact Profile-14*. The COHRA1 protocol utilized the OHIP-14, a self-report instrument comprised of 14 items related to OHRQoL. The original version of the OHIP included 49 items [39] loading onto seven subscales. The OHIP-14 has demonstrated internal consistency (*α* = .90), test-retest reliability (*r* = 0.79–0.94), and construct validity in numerous samples around the world [40–42]. Due to lack of evidence for adequate factorial validity for the OHIP-14, a number of factor structures have been tested via confirmatory factor analyses. Later researchers utilized a stepwise regression analysis to reduce the original OHIP scale to only two items per subscale. The reduced-item scale still accounted for 94% of the variance in the original OHIP, allowing greater ease of administration and clinical utility [43]. More recently, John and colleagues argued that the original OHIP and other OHRQoL assessments can be reduced to four major dimensions (i.e., oral function, orofacial pain, psychosocial impact, and orofacial appearance) [44–46], although the OHIP-14 does not assess the latter factor. Dos Santos et al. [47] proposed the use of a three-factor structure (psychosocial impact, pain and discomfort, and functional limitations) for the OHIP-14 that parallels the John et al. [46] factors, which was utilized in the current study.

### 2.3. Periodontal Screening and Recording Index (PSR)

**Figure 1:**
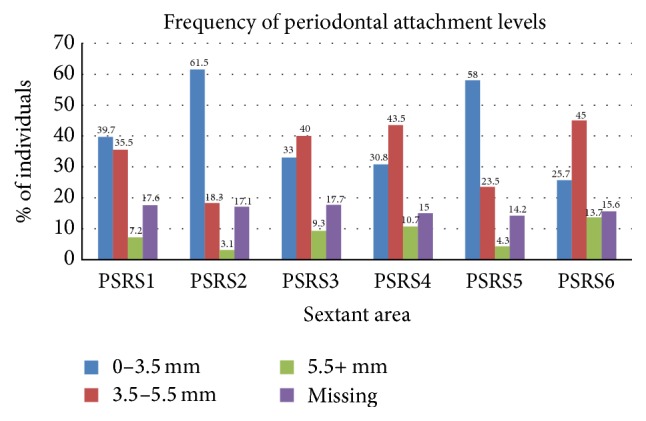
*Note. N* = 1,339 adults.

**Figure 2:**
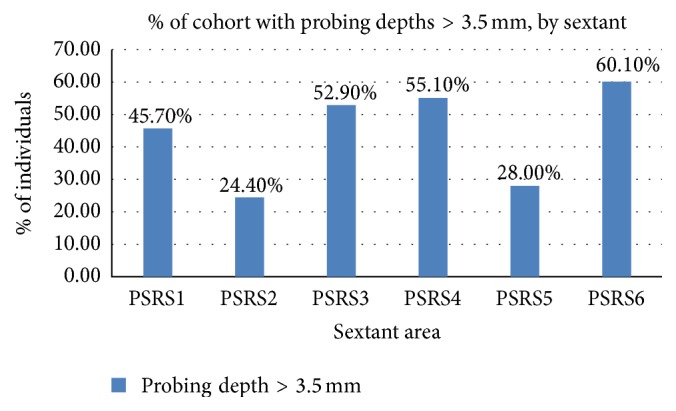
*Note.* Edentulism assumed to be due to periodontal disease, although models with and without this assumption were tested in SEM.

The COHRA1 periodontal screening indicators as well as the training and calibration of examiners were previously described [48, 49]. Modified Periodontal Screening and Recording (PSR) [50] indices were produced through oral health assessments conducted by a licensed and calibrated dentist or dental hygienist. To record probing depths, the mouth was divided into sextants. Gingival crevices around each tooth were examined, and researchers recorded the deepest probing depth found in each sextant. Probing depths were categorized as shallow (i.e., <3.5 mm), moderate (i.e., 3.5 mm–5.5 mm), or deep (i.e., >5.5 mm). If all teeth in a sextant were missing, they were treated in two ways as in Shaffer et al. [49] and Landry and Jean [50]. In one model, these areas were treated as “missing related to periodontal disease” (i.e., treated as having a probing depth of > 5.5 mm) and another model as “missing due to other, nonperiodontal disease factors” (i.e., treated as having a probing depth of < 3.5 mm). Figures 1 and 2 depict the distribution of frequency of probing depths across sextants.

### 2.4. Analytic Approach

In order to test the aforementioned hypotheses, a structural equation modeling (SEM) approach was utilized. Two models—missing due to periodontal disease and missing due to nonperiodontal factors—were tested. SEM includes integrating two major components—a measurement approach based on factor analysis (i.e., confirmatory factor analysis) and a structural path analysis [51]. By combining factor analysis and path analysis, relations between latent (and other) variables can be examined, while taking into account measurement error. More traditional methods of analysis such as analysis of variance (ANOVA) and regression assume all variables and constructs are perfectly measured, often resulting in inaccurate or underestimated coefficients and *p* values [51, 52]. When all else is equal, decreased and accounted for measurement error leads to less biased results via SEM.

*Model Assumptions*. SEM analyses follow certain assumptions, including that variable relations are linear, arise from independent observations, have residuals that are normally distributed, have general equality of variance across variables, and exhibit no signs of extreme multicollinearity and that missing data are handled appropriately [53, 54]. These assumptions were assessed using histograms and descriptive statistics. Assumptions based on linear relations, normality of residuals, and equality of variance were satisfied. Because the COHRA1 data included household information, the independence assumption was possibly violated, but the present models included only adults (18 and older), which reduced the possible consequences due to lack of independence.

In order to account for missing data (beyond the missing due to periodontal issues mentioned earlier), a Full Information Maximum Likelihood method (FIML) in MPLUS 7.4 [55] was incorporated. Previous literature has argued FIML to be more robust in handling missing data than other (e.g., listwise deletion, pairwise deletion, or mean replacement) methods [56]. To assess model fit, specific indices included the root mean square error of approximation (RMSEA), comparative fit index (CFI), Tucker-Lewis index (TLI), and standardized root mean square residual (SRMR). Each of these indices provide unique information and are suspect to various assumptions (e.g., CFI can be inflated due to model complexity), thus the use of all four. Previous researchers suggest heuristic cutoff guidelines for good model fit including a RMSEA value below .06, a CFI and TLI above .90, and SRMR below .08 [57, 58].

*Measurement Approach*. Each of the self-report instruments (FPQ-9, DFS, and OHIP-14) were individually analyzed via confirmatory factor analyses to establish the measurement portion of the model. That is, confirmatory factor analysis (and later combined with multiple regression analyses) allows the measurement error inherent in each variable to be accounted for and provides less biased results [51, 52]. In each case, MPLUS 7.4 [55] modification indices were used to allow for improved model fit (and thereby a reduction in measurement error). The FPQ-9 factor structure tested included the nine items loading onto three subscales which loaded onto a higher order total score as theoretically derived [38].

In order to provide more granular interpretations, the three DFS component subscales were utilized, instead of a total score. Thus, the 19 items (excluding the omnibus item 20) loaded onto their appropriate subscales.

As previously noted, various factor structures have been proposed for the OHIP-14. The present study utilized the three subscales (psychosocial impact, pain and discomfort, and functional limitations) outlined by dos Santos et al. [47]. After an exploratory factor analysis of the current data and testing various factor structures, it was determined that the three-factor solution [47] best fit the data.

**Figure 3:**
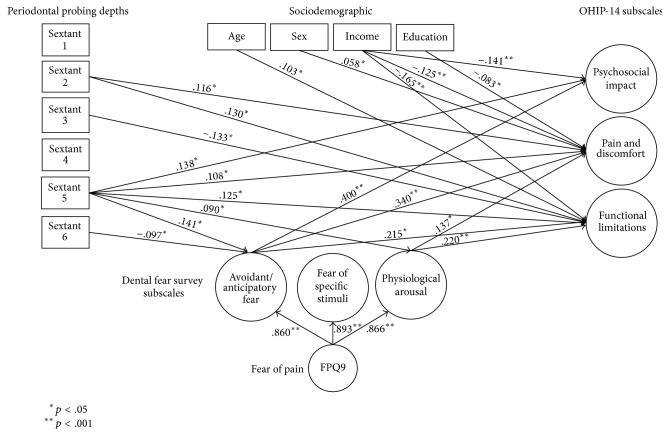
*Note.* Edentulism assumed to be due to periodontal disease in the model.

*Path Analysis (Full SEM)*.  Figure 3 includes the full structural equation model used in this study. Directionality was implied in the model by having OHIP-14 subscales regressing on the periodontal indicators and on the DFS subscales which regressed on the FPQ-9. In order to correct for a nonpositive definite matrix, the mean of the PSR sextant six was fixed to 0.876. Both of the aforementioned hypotheses were tested by first estimating the associations between PSR indicators and OHIP-14 subscales. In addition, in order to test the second hypothesis, the relationship of fear of pain (FPQ-9) and dental fear (DFS subscales) as well as the relationship between the DFS subscales and OHIP-14 subscales were estimated. After results indicated a unique relationship between the mandibular anterior sextant (sextant five), the avoidance and anticipatory fear subscale of DFS, and the OHIP-14 subscales, indirect effect analyses also were utilized. In all cases, there was an alpha cutoff of .05.

## 3. Results

Figure 1 displays the model tested in this study. Confirmatory factor analyses with modification indices are outlined in Table 2. The FPQ-9 fit the data well using the higher order (three subscales loading onto a total fear of pain score) factor structure. The three DFS subscales of avoidant/anticipatory fear, fear of specific stimuli, and physiological fear fit the data by itself as well (RMSEA = .041; CFI = .920; TLI = .911; SRMR = .057). Finally, the three subscale factor structure as described by dos Santos et al. [47] produced good model fit. Although a complex model, the final SEM also produced adequate model fit. Due to only minor differences in the two models—missing due to periodontal issues and missing due to nonperiodontal issues—results from the former model are presented.

### 3.1. Hypothesis 1: PSR Indicators and OHIP-14 Subscales

The full structural equation model included a number of statistically significant results. The probing depths in the maxillary anterior and mandibular anterior regions (sextants two and five) were shown to have significant relationships with OHIP-14 subscales. More specifically, the maxillary anterior sextant was significantly associated with pain and discomfort as it relates to quality of life (*β* = .116, *p* = .017) as well functional limitations (*β* = .130, *p* = .011). The mandibular anterior region had the broadest impact as far as number of significant relationships. This sextant had a significant association with the psychosocial impact subscale of the OHIP-14 (*β* = .138, *p* = .001), with pain and discomfort (*β* = .108, *p* = .019) and with the functional limitations subscales (*β* = .125, *p* = .010). One region, sextant three, was negatively associated with functionality (*β* = −.133, *p* = .020). All significant relationships are portrayed in Figure 1 using solid-line arrows.

### 3.2. Hypothesis 2: Mediating Relationship of Dental Fear

Consistent with prior research, and as expected, the FPQ-9 latent total score was predictive of all three DFS subscales.  Table 3 describes these relations. Regarding the three subscales from the DFS, the fear of specific stimuli did not associate significantly with any of the three OHIP-14 subscales. Physiological responses to dental fear were associated with the pain and discomfort subscale (*β* = .137, *p* = .048) and the functional limitations subscale (*β* = .220, *p* = .011). Some of the largest effects were associated with the avoidant and anticipatory fear subscale. This scale related to the psychosocial OHIP-14 subscale (*β* = .400, *p* < .001) as well as the pain and discomfort subscale (*β* = .340, *p* < .001). Another relationship of significance was between the avoidance and anticipatory fear subscale and the functional limitations scale (*β* = .215, *p* = .018). The avoidance and anticipatory fear subscale also played a noticeable role in the mediation between PSR indicators and OHIP-14 scores. Sextant six was negatively associated with avoidance and anticipatory fear (*β* = −.097, *p* = .048).

As noted, the main effect of the mandibular anterior sextant on the pain and discomfort subscale was statistically significant (Main effect *β* = .108, *p* = .019). The total mediating effect via the sextant-avoidance-pain pathway also was significant (indirect effect via avoidance *β* = .048, *p* = .007; total effect *β* = .165, *p* = .001). In addition, there was a mediating effect associated with the mandibular anterior sextant, avoidance and anticipatory fear, and the psychological impact related to quality of life (main effect *β* = .138, *p* = .001; indirect effect via avoidance *β* = .056, *p* = .006; total effect *β* = .199, *p* < .001). The total effects of the mandibular anterior sextant and the quality of life subscales were also mediated by the physiological arousal DFS subscale. These specific indirect effects, although not statistically significant, contributed to the overall total indirect effect of this particular sextant on the pain and discomfort as well as the functionality subscales of the OHIP-14. In the model in which edentulous sextants were not assumed to be due to periodontal issues, the *p* value for the direct effect between the physiological arousal subscale and the pain and discomfort subscale changed from *p* = .048 to *p* = .056. The overall indirect effect was, however, still significant.

## 4. Discussion

The current study examined the relations among periodontal disease indicators, fear of pain, dental fear, and oral health-related quality of life. The role of avoidant behavior and anticipatory fear, as a component of dental care-related anxiety and fear, emerged as a significant factor that impacts OHRQoL. Previous research has indicated the association of periodontal disease measures with impaired OHRQoL [18, 19]. The present approach, however, added more granular information which helps to explain which regions of the oral cavity may be most associated with OHRQoL, at least in early stages of periodontal involvement. Additional research should further examine these relations and the mechanisms that may be involved in quality of life. Also notable were the findings that the anterior regions of the mouth, and in particular the mandibular anterior sextant, play interesting roles in affecting quality of life related to oral health.

The present study confirmed the importance of fear of pain in dental fear. Individuals who may have high indication of periodontal disease in the mandibular anterior area and high levels of fear of pain also may also have especially high levels of avoidant behavior and anticipatory fear related to oral healthcare. This in turn may have a compounding effect on these individuals' quality of life as it relates to oral health. Future studies and interventions could target reducing fear of pain and modification of avoidant behavior in individuals with indicators of periodontal disease and assess the impact on OHRQoL.

The role of components of dental care-related anxiety and fear as mediators between periodontal disease indicators and quality of life is interesting in light of the previous research by Ng and Leung [28]. Indicators of periodontal disease may be easier for individuals to ignore in the posterior areas of the oral cavity, but once they are evident in the anterior regions, they are more difficult to deny, which may lead to increased dental fear, due to the knowledge that professional dental care is imperative. Fear of pain is a compounding factor in such instances, of course, as periodontal disease may be associated with pain, and anticipation of necessary professional care may lead to fears about experiencing pain during procedures such as scaling and root planning.

Although not a statistically significant indirect effect, part of the total mediating effect between sextant five and quality of life was due to the physiological arousal DFS subscale. In particular, physiological arousal mediated and had a direct effect on the pain and discomfort OHIP-14 subscale as well as the functional limitation subscale. Future interventions and subsequent research could be aimed at reducing the physiological arousal that often accompanies dental fear, fear of pain, and even the manifestation of pain itself. By reducing fear responses, individuals with possible periodontal disease may be less likely to avoid treatment or may tolerate treatment more easily due to the reduction in physiological activation.

These findings have clinical relevance in terms of the importance of fear and pain and their interaction, in oral health care settings [59, 60]. Past research has indicated a need for proper training in anesthetic techniques for dentists [61] and an interest in learning about the most effective techniques [62]. Updates of topical and local anesthetic agents are available, along with newer strategies for delivering local anesthetics [63–65].

Boyce and colleagues [63] suggest that fear and anxiety continue to be the main reason patients avoid or do not follow up with dental care. They suggest that oral health care professionals have the responsibility of educating patients on the many advances to minimize pain and sharing how the developments in anesthesia practiced in dentistry can help alleviate both pain and anxiety. These pharmacological advances have implications for periodontal disease and its treatment.

## 5. Limitations

Results must be understood in light of the limitations in the study design. First, the periodontal phenotype was based on measurement of a PSR index, which is a nonclinical measure related to periodontal disease. Because the PSR indexing used pocket depth to estimate attachment loss rather than measuring attachment directly, it may underestimate attachment loss especially in older patients with severe gingival recession [50]. Given that the present population is relatively young, however, severe gingival recession is unlikely to be a common problem. Thus, estimates based on probing depth may be a good surrogate for disease activity [48]. In terms of measurement, the choice of the OHIP-14 did not allow for a measure of orofacial appearance, which may be important in terms of the visibility of the two sextants that related to OHRQoL in this study.

While the sample is a unique one from north central Appalachia, it does not necessarily represent the entirety of the diverse Appalachian region. Reports of avoidance behavior were gleaned from a DFS subscale, but no actual dental attendance data were incorporated. Future research might explore how specific dental appointment attendance may mediate the relation between periodontal disease and OHRQoL. The present analyses did not account for possible clustering due to adults living in the same household. As a result, the relationships are probably unbiased, but the inferences about these relationships (e.g., beta coefficients and *p* values) could be inflated. In addition, even with 1,339 participants, with as complex a model as was included in this study, it is possible that the study was underpowered and that other relationships could have been otherwise detected.

## 6. Conclusions

The current study was designed to examine associations between periodontal status and oral health-related quality of life and to test the mediating effects of dental fear as influenced by fear of pain. The resulting evidence suggests that there are unique relationships between the maxillary anterior and mandibular anterior sextants and OHRQoL. In addition, when accounting for fear of pain, aspects of dental fear mediate the relationship between periodontal measures and OHRQoL, especially via avoidant behavior and anticipatory fear. These results merit further investigation into clinical applications and how pharmacological and behavioral interventions for fear of pain and dental fear may encourage patients with periodontal disease to seek treatment early and thereby improve their overall quality of life.

## Figures and Tables

**Table 1:** Sample demographics.

	*M*/*N*	SD/%
Age	34.34	9.52
Sex		
Men	487	36.37%
Women	852	63.63%
State of residence		
West Virginia	881	65.80%
Pennsylvania	458	34.20%
Income		
less than 10,000	269	20.10%
10,000 to 14,999	203	15.20%
15,000 to 24,999	224	16.70%
25,000 to 34,999	157	11.70%
35,000 to 49,999	158	11.80%
50,000 to 74,999	81	6.00%
75,000 to 99,999	38	2.80%
100,000 to 149,999	19	1.40%
150,000 to 199,999	2	0.10%
200,000 or more	6	0.40%
Missing	182	13.60%
Education		
No high school diploma	190	14.2%
High school diploma/GED	556	41.5%
Technical school	171	12.8%
Some college, no degree	161	12.0%
Undergraduate degree	131	9.80%
Graduate degree	63	4.70%
Missing	67	5.00%

**Table 2:** Model fit indices produced by confirmatory factor analyses.

Model	RMSEA	CFI	TLI	SRMR
Fear of Pain Questionnaire-9 (FPQ-9)	0.063	0.973	0.951	0.033
Dental fear survey (DFS)	0.051	0.961	0.954	0.051
Oral Health Impact Profile-14 (OHIP-14)	0.055	0.958	0.944	0.036

*Note*. The DFS factor structure did not include the higher order total score but rather the individual items loading onto the three subscales.

**Table 3:** Relationships between FPQ-9 total scores and the three DFS subscales.

FPQ-9 total score as a predictor	*β*	SE	Est./SE	*p*
Avoidant/anticipatory fear	0.860	0.019	46.347	<.001
Fear of specific stimuli	0.893	0.014	64.465	<.001
Physiological fear	0.866	0.018	47.504	<.001

*Note*. Standardized beta values are according to Mplus 7.4 STDYX standardization.
